# Supplementary material for: Shifting from Ammonium to Phosphonium Salts: A Promising Strategy to Develop Next-Generation Weapons against Biofilms
Source: Pharmaceutics. 2024 Jan 5;16(1):80. doi: 10.3390/pharmaceutics16010080 (PMC10819902; doi:10.3390/pharmaceutics16010080)
Supplement: Supplementary file 1 [file pharmaceutics-16-00080-s001.zip › pharmaceutics-2764665-supplementary.pdf]

Supplementary Materials

Table S1. Clinically relevant MDR pathogens with the associated infections [7].

Bacteria	No longer functioning Drugs	Infections	Ref.
P. aeruginosa	Aminoglycosides, quinolones<br>β-lactams, carbapenems	Cystic Fibrosis<br>COPD	[8]
Enterococcus spp	β-lactams, aminoglycoside<br>Tetracycline, erythromycin<br>Vancomycin, streptomycin<br>Linezolid, quinolones	Endocarditis, UTI, BSI, IAA, IPA	[9,10]
A. baumannii	β-lactams, tetracyclines<br>Quinolones, polymixins<br>Aminoglycosides<br>Lincosamides<br>Carbapenems	HAP, VAP, UTI<br>Meningitis<br>Bacteraemia, GIT<br>SI, WI	[11]
E. coli	Cephalosporins, fluoroquinolones<br>Levofloxacin, TSX	UTI, BSI	[12]
K. pneumoniae	Cephalosporins, carbapenems	Pneumonia, BSI, UTI	[13]
S. aureus	Methicillin	WI, BSI	[14]
Streptococcus pneumoniae	Penicillin	Pneumonia, meningitis otitis	[15]
Nontyphoidal Salmonella	Fluoroquinolones	Foodborne diarrhea, BSI	[16]
Shigella spp	Fluoroquinolones, ampicillin, TSX<br>Nalidixic acid	Diarrhea *	[17]
Neisseria gonorrhoeae	Cephalosporins, tetracyclines<br>Macrolides, TSX, quinolones.	Gonorrhea	[18]
Mycobacterium tuberculosis	Rifampicin, isoniazid, fluoroquinolone	Tuberculosis	[19]
**Fungi**			
Candida spp	Fluconazole, echinocandins	Candidiasis	[20]
Cryptococcus neoformans	Fluconazole	Cryptococcosis	[21]
Aspergillus spp	Azoles	Aspergillosis	[22]
Scopulariopsis spp<br>*Onychomycosis*	Amphotericin B, flucytosine, azoles	Onychomycosis	[23]
**Virus**			
Cytomegalovirus (CMV)	Ganciclovir, foscarnet	AIDS and oncology patients	[24]
Herpes simplex virus (HSV)	Acyclovir, famciclovir, valacyclovir	Herpes simplex	[25]
Human immunodeficiency virus (HIV)	Antiretroviral drugs	AIDS	[26]
Influenza virus	Amantadine, rimantadine<br>Neuraminidase inhibitors	Influenza	[27]
Varicella zoster virus	Acyclovir, valacyclovir	Chicken pox	[25]

Hepatitis B virus (HBV)	Lamivudine	Hepatitis B	[28]
**Parasite**			
Plasmodia spp	Chloroquine, artemisinin, atovaquone	Malaria	[29]
Leishmania spp	Pentavalent antimonials, miltefosine<br>Paromomycin, amphotericin B	Leishmaniasis	[30,31]
Schistosomes	Praziquantel, oxamniquine	Schistosomiasis	[32,33]
Entamoeba	Metronidazole	Amoebiasis	[34]
Trichomonas vaginalis	Nitroimidazoles	Trichomoniasis	[35]
Toxoplasma gondii	Artemisinin, atovaquone, sulfadiazine	Toxoplasmosis	[36-38]

* Bacillary dysentery; TSX = Trimethoprim-sulfamethoxazole; UTI = urinary tract infection; BSI = bloodstream infection; COPD = chronic obstructive pulmonary disease (COPD); IAA = intra-abdominal abscesses; IPA = intra-pelvic abscesses; HAP = hospital acquired pneumonia; VAP = ventilator-associated pneumonia; GIT = gastrointestinal infections; SI = skin infections; WI = wound infections. The references in the Table are available at the end of the main text at the correspondent numbers.

Table S2. General composition of BFs.

**Biofilm Biomass**		
**Organized communities of pathogens' cells (2-5%)**		**Extracellular polymeric substances (EPSs)**
Sessile cells	Persistent cells<br>Dormant cell	Enzymes<br>(polypeptides, 1-60%))
Self-immobilized cells attached to a surface, forming highly coordinated micro-colonies, communicating by the QS system	Small subpopulation of microorganisms reversibly transformed into slowly growing cells	Polysaccharides (40-95%)<br>(Cellulose, PGA, exopolysaccharide)
		Extracellular DNA and RNA (eDNA, eRNA) (1-10%)
		Glycoproteins
		Glycolipids (1-40%)<br>Allow communication between bacteria<br>Stabilize the 3-D structure of BF
		Water (70-97%)<br>(responsible for the flow of nutrients)

PGA = poly-glucosamine.

Table S3. Stages of the BF life cycle.

Stage	Action	Events	Ref.
**1**	Reversible attachment	Free-floating planktonic cells select a surface for attachment and self-immobilize on it<br>The attachment is not permanent<br>The locomotory tools such as flagella and pili are still maintained	[73]
**2**	Irreversible attachment	Expression of QS signaling molecules<br>Formation of extracellular polymeric material<br>⇓ in flagella reversal rates<br>⇓ in flagella gene expression<br>Cells demonstrate drug tolerance	
**3**	Grow and cells division (Maturation I)	Recruitment of cells from the environment<br>Appearance of cell clusters *<br>Formation of micro-colonies	
**4**	Formation of the 3D-structured mature BF (Maturation II)	Expression of genes needed for the mature (3D) extracellular matrix<br>Water-filled channels formation for transport of nutrients and removing waste materials	
**5**	BF dispersion **	⇓ and degradation of matrix components<br>Detachment of planktonic cells<br>Multiplication and dispersal of motile cells<br>⇑ Drug susceptibility ***	

* Several cells thick embedded in the BF matrix; ** promoted by QS or enzyme action; *** sometime antibiotic insensitivity can be retained; ⇓ = decrease, reduction; ⇑ = increase. The reference in the Table is available at the end of the main text at the correspondent number.

Disclaimer/Publisher's Note: The statements, opinions and data contained in all publications are solely those of the individual author(s) and contributor(s) and not of MDPI and/or the editor(s). MDPI and/or the editor(s) disclaim responsibility for any injury to people or property resulting from any ideas, methods, instructions or products referred to in the content.
